# Superhydrophobic PDMS-pCA@CNWF Composite with UV-Resistant and Self-Cleaning Properties for Oil/Water Separation

**DOI:** 10.3390/ma15010376

**Published:** 2022-01-05

**Authors:** Hanyu Wen, Yu-I Hsu, Hiroshi Uyama

**Affiliations:** Department of Applied Chemistry, Graduate School of Engineering, Osaka University, 2-1 Yamadaoka, Osaka 565-0871, Japan; h_wen@chem.eng.osaka-u.ac.jp

**Keywords:** oil/water separation, environmental friendliness, superhydrophobic, UV-resistant, self-cleaning

## Abstract

Oil separation is crucial for avoiding environmental pollution originating from industrial wastewater and oil spillage; therefore, it is essential to develop techniques for oil separation. Herein, a new membrane with superhydrophilicity was synthesized by a facile, green, and low-cost method. First, cellulose non-woven fabric (CNWF) was modified by poly (catechin) (pCA), which has good antioxidant and antibacterial activities, to make it unaffected by ultraviolet light and to improve the stability of the structure. Then, hydrolyzed polydimethylsiloxane (PDMS) was coated on the pCA@CNWF surface via chemical bonding to make the composite hydrophobic. This durable superhydrophobic fabric can be used to separate various oil/water mixtures by gravity-driven forces with high separation efficiency (over 98.9%). Additionally, the PDMS-pCA@CNWF possesses the advantages of flexibility, high efficiency, and an outstanding self-cleaning performance, and demonstrates significant potential for applications in various environments, even under various harsh conditions, which make it very promising for the treatment of oil pollution in practical applications.

## 1. Introduction

With the development of industrialization, the problem of water pollution has become a major challenge. As one of the three major types of industrial wastewater, oily wastewater is viewed as a serious environmental issue, threatening human life and the global ecosystem [1,2,3,4,5]. Oil/water separation is a green technology that separates oil/water mixtures to achieve waste oil recycling and sewage purification. It is widely used in wastewater treatment, domestic sewage treatment, the petrochemical industry, and other fields [6,7,8,9]. In most oil/water separation processes, the treatment of oily wastewater is inefficient and costly, and various oil/water separation materials are not able to separate every type of oily sewage simultaneously. In recent years, some research progress has been made in oil/water separation through chemical, physical, and biological treatment methods [10,11,12,13]. Although a combination of these methods can meet most of the relevant requirements for the separation of oil/water mixtures, there are also some disadvantages, such as a large floor space requirement, long treatment cycles, high cost, and the possibility of secondary pollution [14,15].

Superhydrophobic surfaces (water contact angles higher than 150°) have been rapidly developed in the past few decades to selectively filter or absorb oil from water [16,17]. Based on this principle, Boinovich et al. [18] prepared a superhydrophobic surface on an aluminum alloy surface by laser treatment and low-surface-energy molecular modification. However, problems can be experienced with commonly used organic ultrafiltration membranes and mesh materials, such as swelling and breakage, which limit their application. A superhydrophilic/underwater superoleophobic PVDF membrane prepared by Nayak et al. [19] has been successfully used to separate oil/water emulsions. However, the repeated use of this type of membrane is limited by the problem of oil contamination, in which, pore blockage is restricted after separation. Meanwhile, high energy consumption, long processing times, low separation efficiency, and the requirement for chemical additives have imposed restrictions on their practical applications [20].

With continuously improving public awareness of environmental protection, economy, and reuse, there is an increasing demand for adsorption materials that can effectively separate oil–water mixtures. As an agricultural waste, cellulose from plants has the advantages of a low price, biodegradability, high fiber strength, and porosity, and has been explored as a new type of adsorption material [21,22]. Wang et al. [23] used cotton fiber as a raw material to prepare Janus cotton cloth, which has a good separation effect on oil slicks and emulsified oil. Pi et al. [24] dipped a cotton fabric in a cross-linkable fluorine-containing copolymer material to prepare a superhydrophobic/superlipophilic cotton fabric. However, owing to the complex preparation process of fluorine-containing chemicals, the resulting fabrics have poor environmental stability, and their applications have been limited. Superhydrophobic cellulose-based materials have many disadvantages. For example, when cotton is subjected to superhydrophobic modification, it is usually susceptible to environmental influences, such as acid and alkali, high temperature, and ultraviolet light, which cause the structure of the product to be unstable and affect its long-term use. Non-woven fabrics are usually obtained from polymer slices, short or filamentary fibers, woven together into net-like structures through an air-laid process, followed by water thorn, acupuncture, or hot rolling reinforcement to finish the fabrication. Owing to the high flexibility, low density, high porosity, and low cost, non-woven fabrics are expected to be a promising high-efficiency material [25,26,27,28].

In this study, cellulose non-woven fabric (CNWF), which has a high porosity and low cost, has been used as the base material. In order to make the material unaffected by ultraviolet light, the surface of the CNWF was modified with poly (catechin) (pCA), because pCA has strong antioxidant and antibacterial properties [29] and can be synthesized through a simple method (laccase-catalyzed polymerization). Subsequently, the superhydrophobic material PDMS-pCA@CNWF was obtained by the condensation reaction between modified CNWF (pCA@CNWF) and hydrolysis PDMS. The morphology, surface chemical structure, and composition of CNWF, pCA@CNWF, and PDMS-pCA@CNWF were characterized by scanning electron microscopy (SEM), Fourier-transform infrared spectroscopy (FTIR), and X-ray photoelectron spectroscopy (XPS). The separation efficiency and mechanical durability of the PDMS-pCA@CNWF composite were also investigated. This superhydrophobic material could play an important role in oil/water separation in the future.

## 2. Materials and Methods

### 2.1. Materials

Cellulose non-woven fabrics (CNWF) were purchased from Futamura Chemical Co., Ltd. (Aichi, Japan). Catechin, 2,2-diphenyl-1-picrylhydrazyl (DPPH) reagent, dimethylformamide (DMF), and polydimethylsiloxane PDMS were purchased from Sigma-Aldrich (Tokyo, Japan). Laccase derived from Myceliophthora was kindly donated by Novozymes Japan Ltd. (Chiba, Japan) Chloroform, tetrahydrofuran (THF), hydrochloric acid (HCl), sodium hydroxide (NaOH), and Oil Red O were purchased from Fujifilm Wako Pure Chemical Corporation (Tokyo, Japan). Dimethylformamide (DMF) was purchased from Nacalai Tesque Inc. (Kyoto, Japan). Methylene blue was purchased from Tokyo Chemical Industry Co., Ltd. (Tokyo, Japan). Deionized water (DI water) was used for solution preparation and dilution. All reagents were of analytical grade and used as received without further purification.

### 2.2. Enzymatic Polymerization of Catechin

Catechin (0.15 g) and laccase solution (1000 units·mL^−1^) were added in a mixture of acetone and 0.1 M acetate buffer (pH 5) (total 30 mL), and then placed in a 50 mL flask under air. The mixture was then stirred at 25 °C [30]. After 24 h, polymer precipitates were collected by centrifugation. The product was washed twice with a mixture of water and methanol (95:5, *v*:*v*) and dried at a vacuum oven (EYELA VO5-20150, Tokyo, Japan) to obtain the poly (catechin) (pCA).

### 2.3. Antioxidant Activity of pCA

The DPPH assay is an easy and rapid method for evaluating the antioxidant content of samples. DPPH solution (0.1 mM in ethanol, 2 mL) was mixed with 2 mL of catechin/pCA in ethanol at different concentrations (2, 4, 8, 12,16, and 20 μg mL^−1^) and incubated in the dark for 30 min. The absorbance was measured at 517 nm by using an Infinite 200 plate reader (TECAN, Männedorf, Zürich, Switzerland) [31]. For time-dependent antioxidant measurements, catechin/pCA (20 μg mL^−1^) was added to the DPPH ethanolic solution for different times (2, 4, 6, 8, 10, and 12 h) to measure the absorbance. The % DPPH radical scavenging activity was calculated using Equation (1):

(1) $$Scavenging\;\left(\%\right)=\frac{Abs\left(control\right)-Abs\left(sample\right)}{Abs\left(control\right)}$$

where Abs(control) and Abs(sample) indicate the absorbance of the blank and the reaction solution, respectively.

### 2.4. pCA-Modified CNWF

CNWF samples (6 cm × 6 cm) were cleaned three times with DI water and ethanol to remove impurities and oil from the surface. After drying at 40 °C for 5 h, the cleaned CNWF samples were soaked in pCA solution (2 mg L^−1^, pH 7.0) under stirring at room temperature for 24 h to form a pCA layer on the CNWF surface. The samples were then washed with DI water three times. The pCA-modified CNWF (pCA@CNWF) was obtained after drying at 40 °C for 6 h.

### 2.5. Preparation of Superhydrophobic PDMS-pCA@CNWF

The targeted superhydrophobic PDMS-pCA@CNWF was prepared using a simple dip-coating method. First, PDMS was mixed with THF (10:1, *v*:*v*) and stirred at room temperature to form a homogeneous PDMS solution. Subsequently, the pCA@CNWF was immersed in the PDMS solution for 30 min and cured at 70 °C for 12 h in an oven to complete the PDMS curing. The resultant composite was named PDMS-pCA@CNWF for subsequent experiments.

### 2.6. Oil Adsorption Capacity and Oil/Water Separation Property of PDMS-pCA@CNWF

To determine the oil adsorption capacity of the PDMS-pCA@CNWF, 10 g solvents (THF, DMF, chloroform, and hexane) and 150 mL water were added to a 200 mL beaker, and then 0.05 g of the PDMS-pCA@CNWF was placed in the mixture. After 5 min, the film was taken out [32]. The droplets suspended outside the adsorbent were air-dried. The adsorption capacity was calculated using Equation (2):

(2) $$q=\frac{m_{1}-m_{0}}{m_{0}}$$

where q (g g^−1^) is the adsorption capability of the film, and $m_{0}$ (g) and $m_{1}$ (g) are the weights of the samples before and after adsorption, respectively.

The oil/water separation property of the superhydrophobic PDMS-pCA@CNWF was evaluated using a series of 100 mL 1:1 (*v*:*v*) oil/water mixtures. For better observation, the water and oil phases were colored with methylene blue (MB) and Oil Red O, respectively. Four different organic solvents—hexane, DMF, THF, and chloroform—were selected for the oil phase. The separated liquids were collected to determine the separation efficiency. Each measurement was performed three times and an average was calculated. The separation efficiency (E) of the PDMS-pCA@CNWF for various mixtures was calculated using Equation (3) [33]:

(3) $$E=\frac{m_{1}}{m_{0}}\times 100\%$$

where $m_{0}$ and $m_{1}$ represent the weight of the oil before and after separation, respectively.

### 2.7. Mechanical Durability and Chemical Stability of PDMS-pCA@CNWF

Mechanical durability of the superhydrophobic PDMS-pCA@CNWF was investigated using a sandpaper abrasion test. PDMS-pCA@CNWF was dragged in one direction on sandpaper (20 cm length, 5 cm s^−1^) under a 200 g weight. The water contact angle (WCA) of the samples was measured five times and an average was calculated. WCA of the samples was determined every 10 min.

PDMS-pCA@CNWF samples were immersed in various organic solvents (DMF, THF, hexane, and chloroform) and aqueous solutions with different pH values (pH 1, 5, 7, 9, and 13) for 24 h. After that, the treated PDMS-pCA@CNWF was washed with DI water and then dried in an oven at 80 °C. Subsequently, the chemical stability of each sample was determined by WCAs and SEM images.

### 2.8. Characterization

The pCA powder was analyzed using a Tosoh GPC-8020 apparatus (Tosoh, Tokyo, Japan) with DMF containing 0.10 M LiCl as the solvent, and the flow rate was 1.0 mL min^−1^ at 40 °C. Calibration curves were obtained using poly (ethylene oxide) as the standard. UV spectra of catechin and pCA were recorded on a UV-vis spectrophotometer (U-2810, Hitachi, Tokyo, Japan). FTIR measurements of the samples were recorded in the range of 4000–500 cm^−1^ at a resolution of 1 cm^−1^ using a Nicolet iS5 spectrometer (Thermo Scientific, Waltham, MA, USA) equipped with an iD5 ATR attachment. The surface morphology of the CNWF, pCA@CNWF, and PDMS-pCA@CNWF was observed by SEM (SU3500; Hitachi, Tokyo, Japan) at an accelerating voltage of 15 kV. Before analysis, the CNWF, pCA@CNWF, and PDMS-pCA@CNWF were dried and coated with a gold layer using an MSP-1S (Vacuum Device Inc., Tokyo, Japan). The WCAs of the different PDMS-pCA@CNWF samples were determined using a Drop Master DM300 (Kyowa Interface Science, Saitama, Japan). A water droplet (1.0 mL) was dropped onto the surface of the samples (1 cm × 4 cm), and the contact angle of each film was determined at five different positions. The average values are reported. The surface chemical compositions of CNWF and PDMS-pCA@CNWF were determined using XPS (JEOL JPS-9010MC, Tokyo, Japan). The XPS inert parameters included the power of analysis (wide: 75 W, narrow: 150 W) and monochromatic Al Kα radiation [34]. The survey and high-resolution XPS spectra were obtained at fixed analyzer pass energies of 160 eV and 10 eV, respectively. Peak-differentiation analysis was performed using the CasaXPS Version 2.3.15 computer software.

## 3. Results

### 3.1. Enzymatic Polymerization of Catechin

**Scheme 1:**
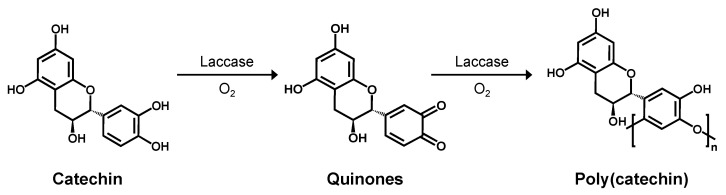
Schematic representation of the polymerization of catechin.

pCA was synthesized through a simple laccase-catalytic method by the polymerization of catechin (Scheme 1), because laccase is highly active for the oxidative polymerization of phenolic compounds [35]. The molecular weight of the pCA was estimated by size exclusion chromatography (SEC), with DMF containing 0.10 M LiCl as the eluent. The number-average molecular weight (Mn) and polymer dispersity index (PDI) were 3419 and 2.0, respectively.

**Figure 1:**
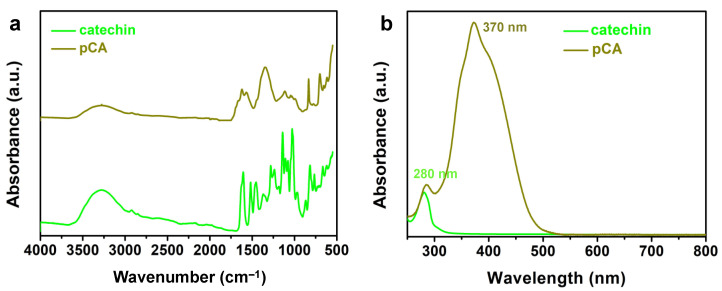
(**a**) FTIR spectra of catechin and pCA powders. (**b**) UV-vis spectra of catechin and pCA in methanol.

Figure 1a shows the FT-IR spectra of catechin and pCA. The peak pattern of pCA is similar to that of catechin, although all of the peaks became broader. In the pCA spectrum, the peak at 3300 cm^−1^ is attributed to the vibration of the O–H linkage of phenolic and hydroxyl groups. The peak at 1570 cm^−1^, distributed to the C=C vibration of the aromatic group, is also observed. According to the literature [36,37], peaks at 3390, 1602, and 1065 cm^−1^ are attributed to the characteristic functional groups of polyflavonoids. The peak at the range of 1310–1390 cm^−1^ distributed to phenol group (–C–OH) deformation vibrations.

The UV-vis spectra of catechin and pCA are shown in Figure 1b. Peaks at 280 nm in both catechin and pCA were due to the π–π * transitions of the aromatic fragment appearing. A new large broad peak appeared in the spectrum of pCA at 370 nm, which indicated the occurrence of catechin polymerization in the enzyme environment [38]. Along with an increase in the basic nature of the reaction environment, the speed and intensity of the appearance of the polymerization product increased.

**Figure 2:**
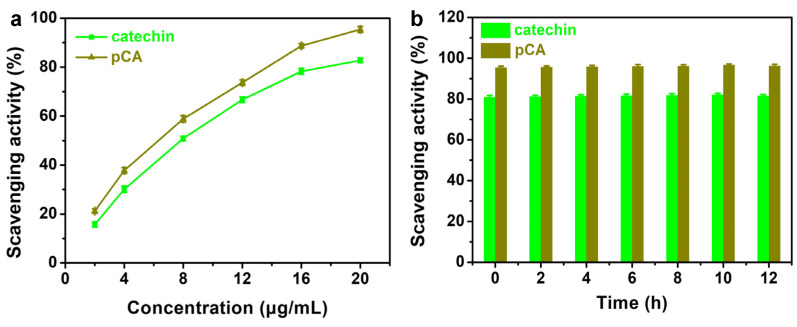
Antioxidant assessment of the catechin and pCA depending on the (**a**) concentration and (**b**) time. Each value represents the mean ± standard deviation (SD) (*n* = 3).

The antioxidant activity of pCA was evaluated using the DPPH method, which is based on the quenching of synthetic-free radicals. In this method, the purple active radical is reduced by antioxidants present in the test sample to a yellow product. As shown in Figure 2a, at a powder weight of 20 μg mL^−1^, the DPPH radical scavenging activity of the pCA was 95%, whereas that of the catechin was 90%. Meanwhile, the DPPH radical scavenging activity of the pCA was consistently higher than that of the catechin, irrespective of the reaction time (Figure 2b). These results suggest that pCA possesses more potent radical scavenging activity than catechin.

### 3.2. Preparation of Superhydrophobic PDMS-pCA@CNWF

**Figure 3:**
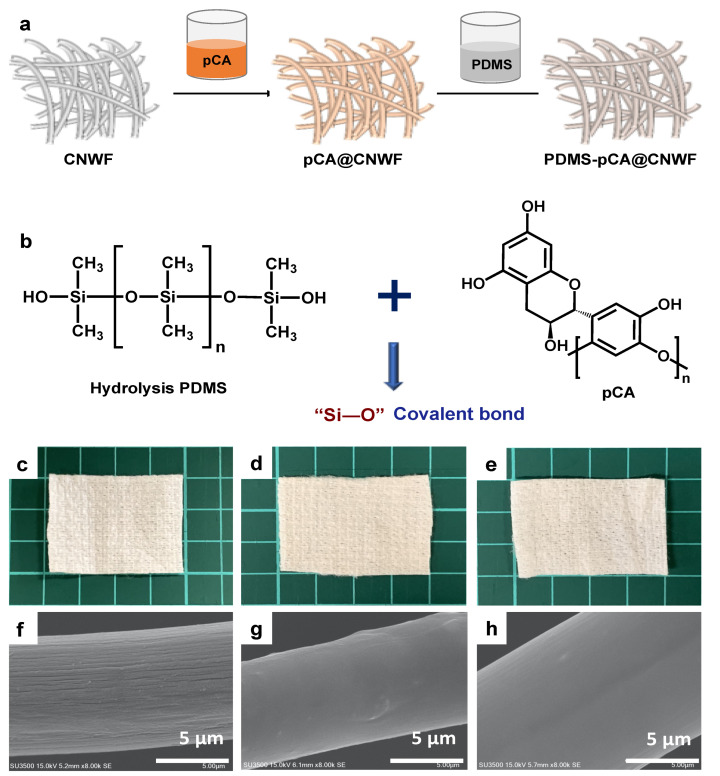
(**a**) Fabrication process of PDMS-pCA@CNWF. (**b**) Mechanism of the reaction between PDMS and pCA@CNWF surface functional groups. Photographs and SEM images of CNWF (**c**,**f**), pCA@CNWF (**d**,**g**), and PDMS-pCA@CNWF (**e**,**h**).

After the successful preparation of pCA, a modified CNWF (pCA@CNWF) with high antioxidant and antibacterial activity was obtained. Then, the targeted superhydrophobic CNWF (PDMS-pCA@CNWF) for oil/water separation was prepared. Subsequently, the silanol groups from hydrolysis PDMS was reacted with the hydroxyl groups from pCA@CNWF to form a covalently bonded and robust hydrophobic layer through condensation (Figure 3a,b).

The successfully obtained superhydrophobic PDMS-pCA@CNWF was confirmed by SEM, FTIR, XPS, and WCA measurements. First, SEM measurements were attituded to characterize the morphological structure of the samples. Figure 3c–h shows the surface morphology of the samples. Figure 3f shows a typical image of the CNWF with an average diameter of approximately 9.1 μm. The pCA@CNWF is shown in Figure 3g, and its diameter is 8.3 μm. The surface morphology of pCA@CNWF changed after being coated with PDMS (Figure 3h), resulting in a diameter of 12.3 μm. PDMS coatings uniformly cover the pCA@CNWF wires, and almost no PDMS is present in the pores, which ensures that water can pass freely through the prepared PDMS-pCA@CNWF.

**Figure 4:**
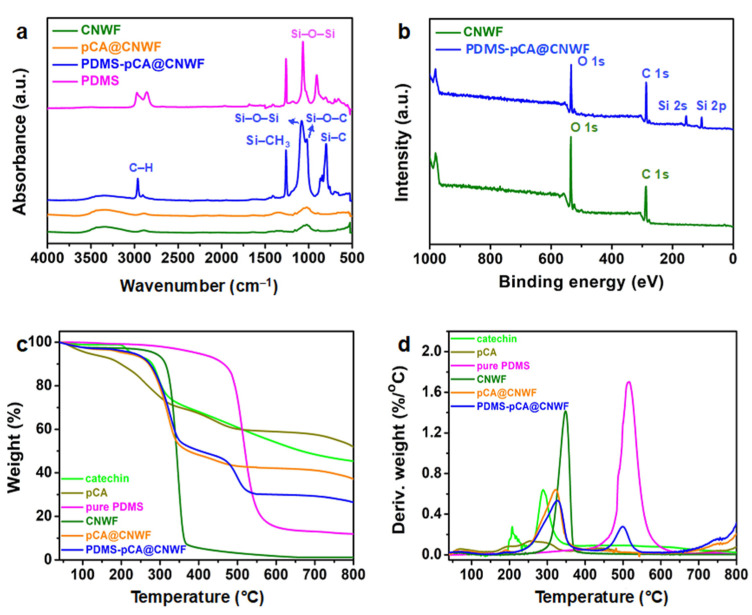
(**a**) FT-IR spectra of PDMS, CNWF, pCA@CNWF, and PDMS-pCA@CNWF. (**b**) XPS survey spectra of CNWF and PDMS-pCA@CNWF. (**c**) TGA and (**d**) DTG curves of catechin, poly (catechin), PDMS, CNWF, pCA@CNWF, and PDMS-pCA@CNWF.

The surface chemical composition of the samples was investigated by FTIR. As shown in Figure 4a, peaks at 1023 and ~3400 cm^−1^ in the spectra of the CNWF and pCA@CNWF are assigned to C–O–C and the O–H stretching vibration, respectively. PDMS-pCA@CNWF shows a peak at 1068 cm^−1^, indicating the existence of Si–O–C bonds, whereas the PDMS spectrum shows a peak at 1081 cm^−1^, which is the Si–O–Si bonds [39]. These results indicate that the hydrolyzed PDMS can be coated on the pCA@CNWF to form the PDMS-pCA@CNWF.

The chemical composition of the resultant PDMS-pCA@CNWF was examined by XPS measurements. As shown in Figure 4b, in addition to peaks for C (284.8 eV) and O (102.3 eV) [40,41], characteristic peaks for Si can be seen for the PDMS-pCA@CNWF spectrum when compared to those of the CNWF. The peaks at 102.3 eV and 152.0 eV are attributable to Si 2p and Si 2s from PDMS [42,43]. Therefore, these results indicate the successful deposition of PDMS on the pCA@CNWF surface.

Thermal stabilities of the samples were also analyzed, as shown in Figure 4c,d; catechin and pCA exhibit similar curves. Lispergure et al. [44] reported that the complex aromatic structure of naturally condensed catechin tannins results in high thermal resistance. The decomposition of Acacia dealbata tannin is almost complete at a temperature of 600 °C, and the remaining weight of tannin is approximately 44%. The weight of pCA at 600 °C was approximately 59%, and that of the reference, catechin, was approximately 54%. Therefore, pCA has a higher thermal stability with a comparison of natural tannin. The weight loss of the CNWF commences at 285 °C and stops at 373 °C, and the weight at 800 °C is near 0%. Compared with pCA@CNWF and PDMS-pCA@CNWF, it can be stated that both pCA@CNWF (37.3%) and PDMS-pCA@CNWF (26.3%) have a higher thermal stability than CNWF.

### 3.3. Oil/water Separation Using PDMS-pCA@CNWF

**Figure 5:**
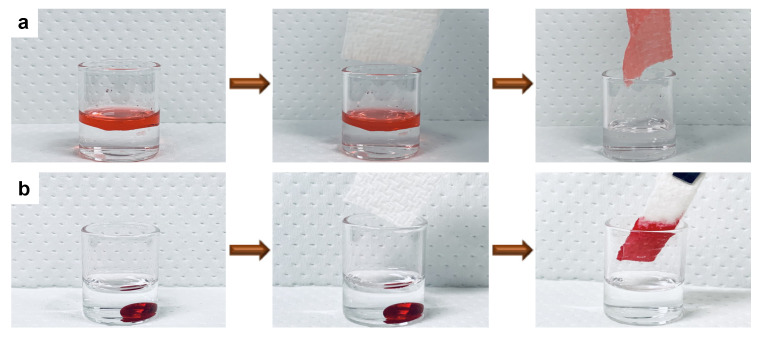
Photographs of selective absorption of the PDMS-pCA@CNWF for Oil Red O-stained (**a**) hexane and (**b**) chloroform in oil/water mixtures.

**Figure 6:**
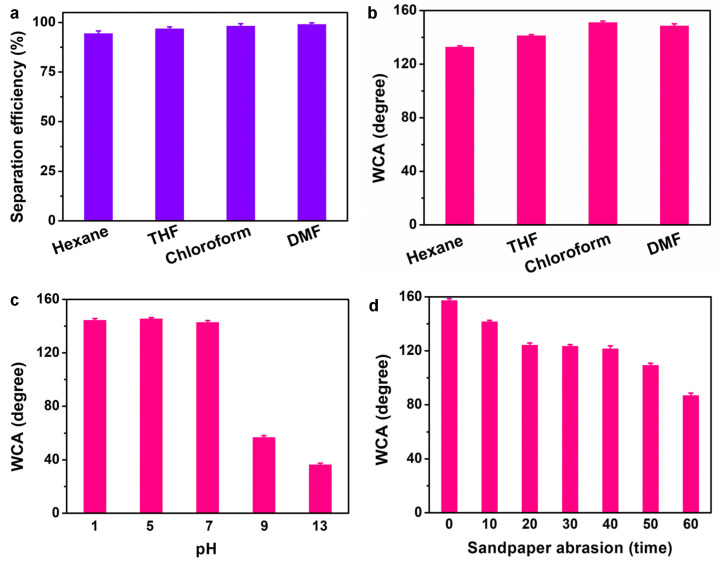
(**a**) Separation efficiency of different oil/water mixtures using superhydrophobic PDMS-pCA@CNWF. (**b**) The water contact angle of different solvents, (**c**) pH, and (**d**) abrasion time using sandpaper of PDMS-pCA@CNWF.

The obtained PDMS-pCA@CNWF exhibited superhydrophobicity, which was indicated in Figure S1. Therefore, it was expected to display excellent oil/water separation. Figure 5 shows that PDMS-pCA@CNWF can selectively adsorb oil solvents from oil–water mixtures containing Oil Red O-stained n-hexane or chloroform. As expected, hexane and chloroform can be completely absorbed by PDMS-pCA@CNWF within a few seconds, as they are superoleophilic and superhydrophobic, leaving clean and transparent water. The same results were also observed for MB-stained hexane and MB-stained chloroform mixtures. The same separation process was tested for various mixtures of DMF/water and THF/water, and the results were as good as those for the chloroform/water mixture. The oil/water separation efficiencies of the samples were calculated and are presented in Figure 6a. The results of the PDMS-pCA@CNWF for the mixtures were always above 94.5%. However, the separation efficiency did not reach 100%, which is mainly attributed to the adsorption capacity of the superhydrophobic PDMS-pCA@CNWF for the oils.

The adsorption capacities of the PDMS-pCA@CNWF for various oil solvents were also measured, as shown in Figure S2. The adsorption capacities for hexane, THF, chloroform, and DMF were 15.4 g g^−1^, 20.6 g g^−1^, 22.4 g g^−1^, and 21.1 g g^−1^, respectively. The comparison with other materials reported for oil/water separation in Table S1 indicates that the PDMS-pCA@CNWF in this study has the highest oil absorption capacity. Thus, it can be concluded from the above results that PDMS-pCA@CNWF can be employed as a new environmentally friendly material to achieve highly effective oil/water separation.

### 3.4. Durability and Self-Cleaning Property of PDMS-pCA@CNWF

To improve the durability of the PDMS-pCA@CNWF, solvent damage and mechanical forces during use were investigated. As an important criterion for practical applications, the long-term superhydrophobic durability of the prepared PDMS-pCA@CNWF was also investigated under various rigorous conditions. The thickness of all of the samples is also summarized in Table S2. There were no significant differences in the thickness of all samples, except for pH 9 and pH 13. PDMS was easily soluble in an alkaline environment; meanwhile, pCA was also unstable under these conditions. Thus, the reaction between PDMS and pCA was disrupted and further affected the surfaces of samples, which resulted in the sample thickness decreasing. The chemical resistance of the PDMS-pCA@CNWF was evaluated by immersing it in an extremely chemical environment. As shown in Figure 6b,c, WCAs were in the range of 142° to 151° after the PDMS-pCA@CNWF samples were immersed in various organic solvents and aqueous solutions with different pH for 24 h, exhibiting desirable superhydrophobicity. Notably, the WCAs of the samples treated with strong alkali solutions and hexane decreased, which can be attributed to the slight hydrolyzation and dissolution of PDMS under these conditions. However, even after exposure to hexane, PDMS-pCA@CNWF was still capable of achieving oil/water separation. To investigate the effect of mechanical properties on the robustness of samples, an abrasion test was conducted. With an increase in the number of abrasion cycles, the WCA of PDMS-pCA@CNWF decreased (Figure 6d). However, it remained at approximately 110°, even after 50 abrasion cycles, exhibiting durable hydrophobicity. It can be concluded from the results of mechanical and chemical durability tests that the superhydrophobic PDMS-pCA@CNWF displayed excellent stability in extremely harsh environments.

**Figure 7:**
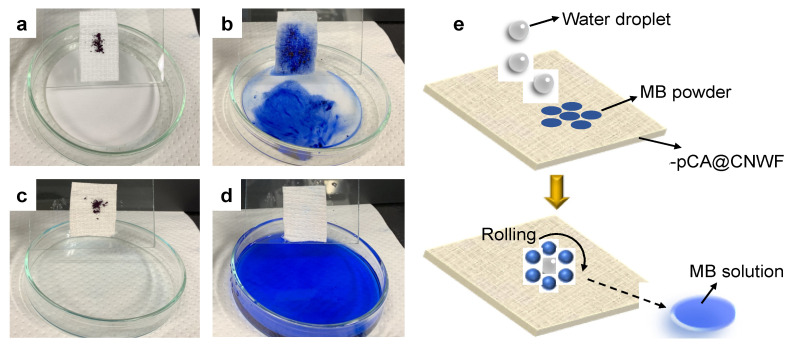
Self-cleaning of MB powder on the surface of (**a**,**b**) CNWF and (**c**,**d**) PDMS-pCA@CNWF. (**e**) Schematic of the self-cleaning activity of PDMS-pCA@CNWF.

The surface self-cleaning properties of PDMS-pCA@CNWF and CNWF were compared. The performance was verified using MB powder as a model contaminant. MB powders are randomly distributed on the surface of the pristine and modified CNWFs, which were tightly adhered to the glass slides (Figure 7a,c). For PDMS-pCA@CNWF (Figure 7d), when waters were dropped to the MB-contaminated sample, the powders were immediately removed and the surface of PDMS-pCA@CNWF remained clean. In contrast, for CNWF, the MB powder immediately dissolved in the water and then contaminated its surface, leaving a blue trace (Figure 7b). A schematic diagram of this process is shown in Figure 7e. The results demonstrate the excellent self-cleaning property of PDMS-pCA@CNWF due to its high water surface tension and low surface energy, which plays an essential role in the anti-fouling behavior.

## 4. Conclusions

In summary, a facile and feasible approach for highly efficient oil/water separation was developed. The antioxidant PDMS-pCA@CNWF with self-cleaning properties enhances UV resistance and is proven as an environmentally friendly material for oil in oil/water mixtures (both light and heavy oils). Owing to the presence of pCA, it has a high antioxidant activity, making it less susceptible to the influence of ultraviolet light and improving its stability. Due to the excellent flexibility of the CNWF, the material can be easily recycled by extrusion. In all cases, the synthetic PDMS-pCA@CNWF has an oil separation efficiency greater than 94% for all types of oil. The WCA of PDMS-pCA@CNWF remained at approximately 110° after 50 abrasion cycles, exhibiting durable hydrophobicity. More importantly, this hybrid material (PDMS-pCA@CNWF) can be applied in a variety of complex environments, particularly in strongly acidic environments, demonstrating its potential application in practical situations. The present strategy provides a simple method to fabricate a novel environmentally friendly superhydrophobic material with a low cost and high efficiency in oil–water mixture separation studies. This research result breaks through the bottleneck of traditional oil/water separation technologies and has broad application prospects.

## Data Availability

The data presented in this study are available within the article.

## Supplementary Materials

The following are available online at https://www.mdpi.com/article/10.3390/ma15010376/s1, Figure S1: Water contact angles of CNWF (a) and PDMS-pCA@CNWF (b). Figure S2: Adsorption capacities of PDMS-pCA@CNWF for different oil solvents. Table S1: Summary of the oil adsorption capacities of various adsorbents. Table S2: Thickness of CNWF, pCA@CNWF, PDMS-pCA@CNWF, and PDMS-pCA@CNWF in the different pH solutions (pH 1, 5, 7, 9, and 13) and various organic solvents (Hexane, THF, Chloroform, and DMF).
