# Supplementary material for: Superhydrophobic PDMS-pCA@CNWF Composite with UV-Resistant and Self-Cleaning Properties for Oil/Water Separation
Source: Materials (Basel). 2022 Jan 5;15(1):376. doi: 10.3390/ma15010376 (PMC8746016; doi:10.3390/ma15010376)
Supplement: Supplementary file 1 [file materials-15-00376-s001.zip › materials-1509948-supplementary.pdf]

materials

Supplementary Material

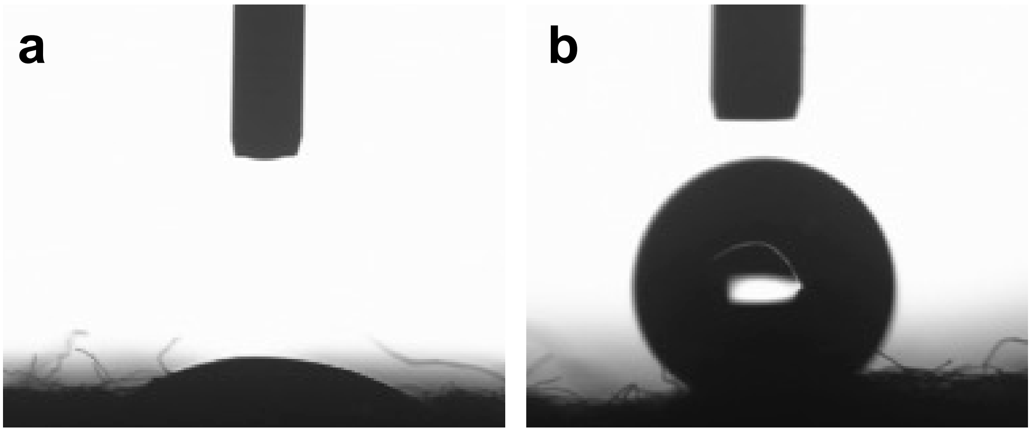

Figure S1. Water contact angles of CNWF (**a**) and PDMS-pCA@CNWF (**b**).

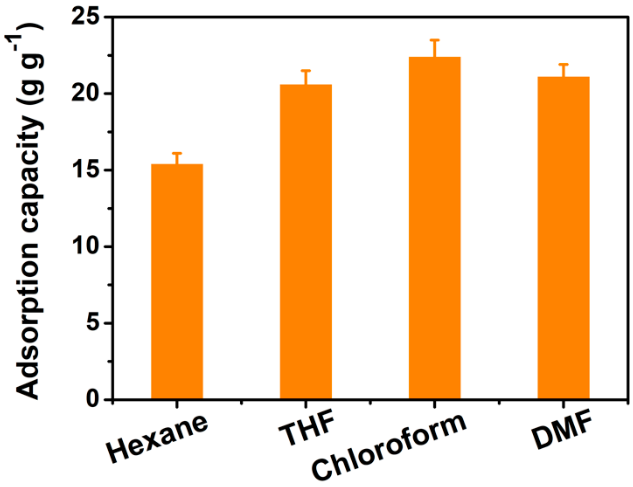

Figure S2. Adsorption capacities of PDMS-pCA@CNWF for different oil solvents.

Table S1. Summary of the oil adsorption capacities of various adsorbents.

Samples	Oil Adsorption Capacity (g/g)	Ref.
Polydivinylbenzene nanofibres	7.5	[1]
Alkenylsuccinic anhydride modified wood-based fibers	6	[2]
Cotton fibers grafted with dialkyl substituted silyl ethers	18	[3]
Polyvinyl alcohol nanofiber membranes	12.7	[4]
Raw cotton fiber	15	[5]
PDMS-pCA@CNWF	22.4	This work

Table S2. Thickness of CNWF, pCA@CNWF, PDMS-pCA@CNWF, and PDMS-pCA@CNWF in the different pH solutions (pH 1, 5, 7, 9, and 13) and various organic solvents (Hexane, THF, Chloroform, and DMF).

Samples	Thickness[a] (μm)
CNWF	1066 ± 2.1
pCA@CNWF	1059 ± 3.0
PDMS-pCA@CNWF	1068 ± 3.5
PDMS-pCA@CNWF (Hexane)	1027 ± 3.8
PDMS-pCA@CNWF (THF)	1049 ± 4.1
PDMS-pCA@CNWF (Chloroform)	1058 ± 2.1
PDMS-pCA@CNWF (DMF)	1056 ± 2.3
PDMS-pCA@CNWF (pH 1)	1051 ± 2.5
PDMS-pCA@CNWF (pH 5)	1055 ± 2.6
PDMS-pCA@CNWF (pH 7)	1049 ± 2.7
PDMS-pCA@CNWF (pH 9)	887 ± 3.1
PDMS-pCA@CNWF (pH 13)	721 ± 4.6

[a]Values are shown as the mean ± standard deviation (SD) (n = 3).

References

1. Zhu, X.; Wang, X.; Liu, Y. Efficient adsorption of oil in water by hydrophobic nonwoven fabrics coated with cross-linked poly-divi-nylbenzene fibers. *J. Chem. Technol. Biotechnol.* **2019**, *94*, 128–135.
2. Payne, K.; Jackson, C.; Aizpurua, C. Oil spills abatement: Factors affecting oil uptake by cellulosic fibers. Environ. *Sci. Technol.* **2012**, *46*, 7725–7730.
3. Jarrah, K.; Hisaindee, S.; Al-Sayah, M. Preparation of oil sorbents by solvent-free grafting of cellulose cotton fibers. *Cellulose*, **2018**, *25*, 4093–4106.
4. Ge, J.; Wu, G.; Yoon, S. Study on the Preparation and Lipophilic Properties of Polyvinyl Alcohol (PVA) Nanofiber Membranes via Green Electrospinning. *Nanomaterials*, **2021**, *11*, 2514.
5. Liu, F.; Ma, M.; Zang, D. Fabrication of superhydrophobic/superoleophilic cotton for application in the field of water/oil separation. *Carbohydr. Polym.* **2014**, *103*, 480–487.
